# Fiber Bragg Grating Monitoring of Thermal Dynamics in Immersion-Cooled Lithium-Ion Batteries

**DOI:** 10.3390/s26216988

**Published:** 2026-11-05

**Authors:** Jiabao Xie, Kuai Yu, Tianfeng Hu, Qingrui Yan, Jiadong Zhang, Wenguo Zhu, Yongchun Zhong, Jianhui Yu, Yihua Qian, Yaohong Zhao, Huadan Zheng, Tuan Guo

**Affiliations:** 1 Key Laboratory of Optoelectronic Precision Measurement and Instruments, Jinan University, Guangzhou 510632, China; xjb2021103764@stu2021.jnu.edu.cn (J.X.); yukuai@stu2023.jnu.edu.cn (K.Y.); tianfenghu@stu2023.jnu.edu.cn (T.H.); 531465375yqr@stu2024.jnu.edu.cn (Q.Y.); 358582a@stu2024.jnu.edu.cn (J.Z.); zhuwg88@163.com (W.Z.); ychzhong@jnu.edu.cn (Y.Z.); jianhuiyu@jnu.edu.cn (J.Y.); tuanguo@jnu.edu.cn (T.G.); 2 Guangdong Key Laboratory of Electric Power Equipment Reliability, Electric Power Research Institute of Guangdong Power Grid Co., Ltd., Guangzhou 510080, China; qianyh2001@163.com

**Keywords:** fiber Bragg grating, liquid immersion cooling, natural air cooling, lithium-ion battery

## Abstract

Dielectric immersion provides a direct thermal boundary for lithium-ion battery abuse tests, while passive fiber Bragg grating (FBG) sensing enables surface-temperature monitoring within the liquid. We monitored fully charged NCR18650BD cells during external short circuits under natural air and static transformer-oil immersion through two 1 km single-mode fiber leads. A seven-point temperature calibration extended to 132.3 °C yielded a sensitivity of 8.76 pm °C^−1^ (R^2^ = 0.99955). In the original tests, peak surface temperatures were 132.3 °C in air and 51.1 °C under immersion, with post-peak half-decay times of 579 and 161 s, respectively. Additional tests reproduced the lower temperature excursion and shorter post-peak half-decay under immersion, although the recorded short-circuit currents differed between cooling conditions. The results characterize these thermal responses and demonstrate the feasibility of electrically passive, long-reach FBG monitoring during immersed battery-abuse experiments.

## 1. Introduction

Lithium-ion batteries play a central role in electric mobility and energy storage, where electrical faults can impose intense thermal loads on individual cells and modules. During an external short circuit, a low-resistance path produces high current, ohmic heating, and polarization losses that elevate the cell temperature. As heating progresses, the evolving electrochemical state and thermal boundary conditions determine the magnitude and duration of the temperature excursion. The transient temperature profile—from rapid heating to the peak and subsequent relaxation—therefore provides a direct basis for assessing the thermal consequences of electrical abuse [1,2,3,4,5,6,7,8,9,10]. Earlier external-short-circuit experiments on cylindrical cells and battery modules have shown that fault resistance, state of charge, and test conditions influence the coupled current and temperature trajectories [11,12].

Immersion cooling is a battery thermal-management approach in which an electrically insulating liquid contacts the cell surface directly and serves as a heat-transfer medium. Its thermal performance depends on the liquid properties and flow conditions. Reviews summarize available immersion coolants and their applications [13,14,15], while experimental studies use fluorinated liquid [16], synthetic ester [17], and modified fluorinated liquid in static battery-module immersion [18]. Vikram et al. [19] experimentally compared natural air cooling with partial and full transformer-oil immersion during battery-module operation. Wang et al. [20] examined the effects of immersion-liquid type and depth on thermal-runaway heating and gas release, while Kannan et al. [21] investigated thermal-runaway and off-gassing behavior of immersed 18,650 cells. These studies concern distinct operating or thermal-abuse conditions and provide context for the present external-short-circuit measurements rather than directly matched benchmarks.

During an external short circuit, heat generation and heat dissipation both evolve with time. Comparing the full surface-temperature trajectories under air and static liquid immersion can reveal differences in peak temperature, heating evolution, and post-peak recovery, while the electrical loading must also be considered.

Surface-temperature sensing in dielectric immersion has practical instrumentation requirements: the sensing element must remain electrically passive and function while immersed, and the readout equipment should be kept away from high-current fault connections. Koshkouei et al. [22] evaluated thermocouple selection and its influence on temperature monitoring of lithium-ion cells. Reviews of battery-monitoring approaches [23,24] discuss a range of sensing modalities, whereas Chen et al. [25] specifically review FBG-based lithium-ion battery safety monitoring, including single- and dual-parameter sensing and applications to temperature, strain, and pressure. General reviews of optical-fiber and FBG sensors [26,27,28] describe their operating principles, design options, and limitations. Reviews of lab-on-fiber and functional optical-fiber diagnostics [29,30] further show how optical fibers can probe physical and chemical changes during battery operation. Together, these works establish the broader role of optical sensing, while also showing that individual studies need to be distinguished by the parameter measured, sensor location, and operating condition.

Several studies have directly applied FBGs to battery temperature measurement. Yang et al. [31] integrated FBG sensors into lithium batteries, calibrated them against co-located thermocouples, and monitored normal and abnormal operation; temperature variations as small as 0.1 °C were detected, and the dynamic response was compared with that of thermocouples. Novais et al. [32] used four FBG sensors to monitor internal and external temperatures during galvanostatic cycling from 1C to 8C, including positions in the active-area center and near a tab-electrode connection, demonstrating the value of internal measurements for resolving temperature gradients. Nascimento et al. [33] compared fiber sensors with thermocouples at multiple battery locations under charging, discharging, and abusive conditions, reporting a faster rise response for the FBG sensors. Nascimento et al. [34] extended the approach to a 37-FBG network for spatial and temporal thermal mapping of a three-cell prismatic lithium-polymer battery pack and identified inter-cell hot spots near a positive tab collector. Liu et al. [35] demonstrated operando temperature monitoring using implanted femtosecond-laser-inscribed optical-fiber sensors. For distributed or remote measurements, Kim et al. [36] combined FBGs with optical frequency-domain reflectometry for physically separated or rotating electric-vehicle systems, Huang et al. [37] embedded FBGs for internal-temperature monitoring and compared the measurements with a thermal model, and Wang et al. [38] used an ultra-weak FBG array to increase the number of monitored temperature points across a battery pack. Collectively, these studies show a progression from local temperature tracking to internal sensing, thermal mapping, and remote multi-point monitoring. Peng et al. [39] measured external-electrode temperature in LiFePO4 batteries using metal-ring-mounted FBGs and PT100 comparisons. Wu et al. [40] reported long-term monitoring of internal temperature fields in high-rate hardcase lithium-ion batteries using embedded FBG sensors. Huang et al. [41] incorporated optical-fiber Bragg sensors into cylindrical cells and used tailored fiber configurations to distinguish temperature and pressure responses. Their sensor placement, cell conditions, and monitored parameters differ from those of the present surface-contact, external-short-circuit experiment.

A related group of studies addresses strain sensing and temperature–strain cross-sensitivity. Sommer et al. [42] used fiber-optic strain sensors to observe fast and slow ion-diffusion-related deformation in pouch cells during cycling. Nascimento et al. [43] developed a hybrid optical-fiber sensing network to discriminate internal strain from temperature, while Matuck et al. [44] used polarization-maintaining FBGs to separate these parameters in 18,650 cells. Their later customized birefringent sensors enabled multi-point measurements of temperature and radial strain [45]. These studies demonstrate that a grating response can contain both thermal and mechanical contributions, and that sensor installation or multi-parameter designs influence interpretation. They therefore provide relevant context for reporting how a surface-contact FBG is mounted and for acknowledging any residual strain contribution that cannot be independently quantified. Xi et al. [46] used embedded optical-fiber sensors to simultaneously monitor internal temperature and strain in solid-state batteries, and Gan et al. [47] demonstrated FBG-array-based temperature and strain monitoring with dual-parameter decoupling in a battery module. These examples reinforce the need to consider strain-induced wavelength shifts when interpreting a single FBG signal. The contact mounting in the present work was intended to limit direct strain transfer; residual contributions remain unquantified without an independent strain measurement.

Fiber-optic battery diagnostics also extend beyond temperature and strain. Mei et al. [48] inserted a compact lab-on-fiber sensor into commercial 18,650 cells to monitor internal temperature and pressure effects during thermal runaway, including pressure features associated with venting and runaway onset. Wang et al. [49] used plasmonic fiber-optic sensing to monitor interfacial ion activity in aqueous batteries, and Han et al. [50] used tilted FBGs to track lithium-ion transport and dendrite growth near lithium-metal electrodes. These studies demonstrate the breadth of operando fiber sensing, but their primary targets include internal pressure, ion transport, and dendrite formation rather than a comparison of cell-surface temperature transients under different cooling boundaries.

Taken together, the literature demonstrates that FBG and other optical-fiber sensors are already used for battery temperature, strain, internal-state, and electrochemical monitoring. Many thermal studies focus on cycling, spatial mapping, internal sensing, or temperature–strain discrimination, while other operando studies target thermal runaway or electrochemical mechanisms. The present work addresses a narrower experimental question: how the local surface-temperature trajectory of a fully charged 18,650 cell evolves during an external short circuit under natural air cooling versus static transformer-oil immersion. We use a passive transmission-mode FBG interrogated through two 1 km single-mode-fiber leads and compare peak temperature rise, post-peak half-decay, and integrated temperature rise across two experimental rounds. The contribution is the combination of a time-resolved external-short-circuit thermal comparison with a long-reach, electrically passive sensing configuration for an immersed abuse test. Because the recorded current profiles differed between cooling conditions in the additional round, the results are interpreted as configuration-level observations rather than a matched-load isolation of the coolant effect.

## 2. Materials and Methods

### 2.1. FBG Temperature-Sensing Principle and Optical System

**Figure 1:**
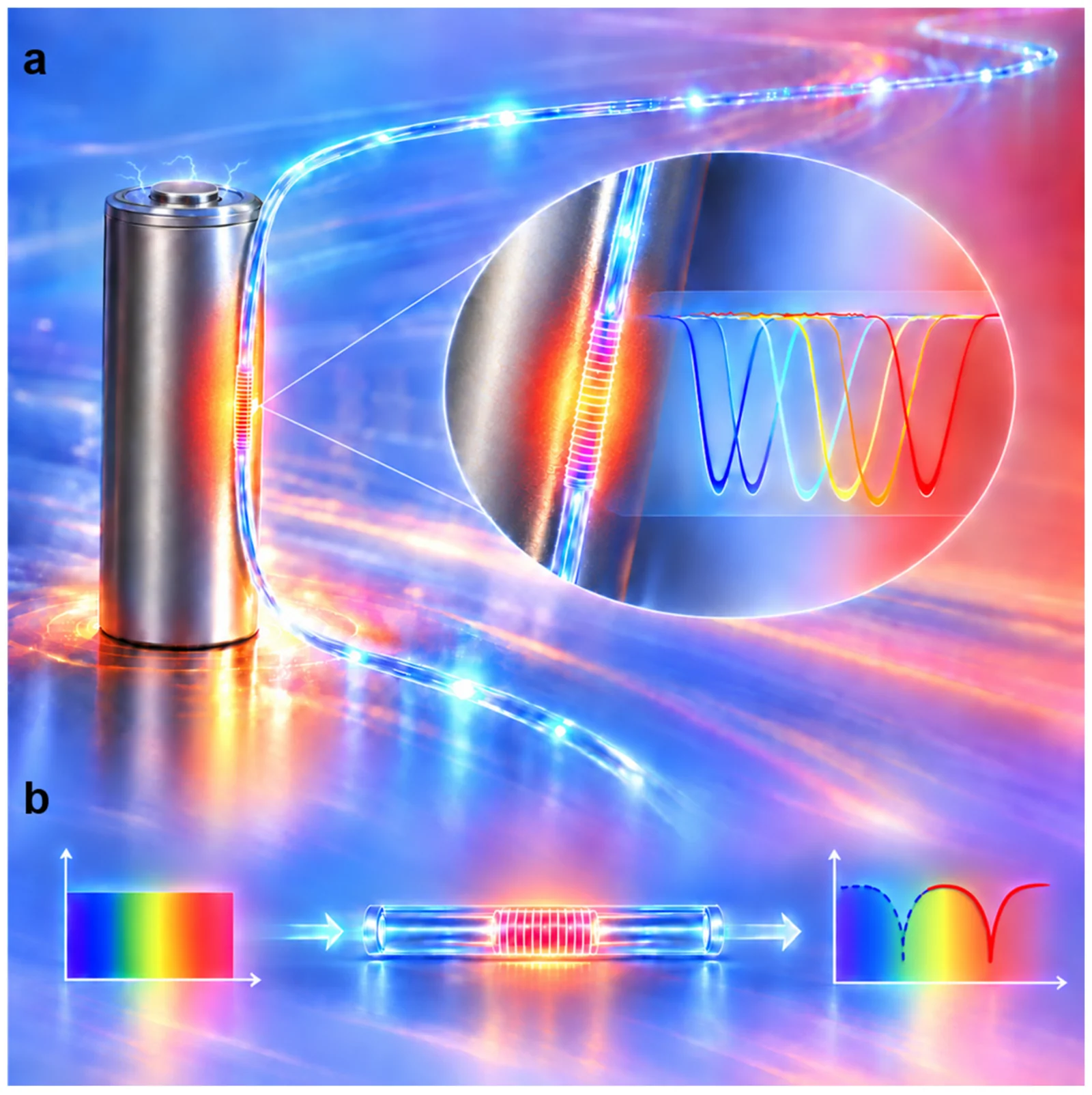
FBG sensing concept and temperature-encoded spectral response. (**a**) Conceptual remote sensing of a battery-mounted grating. (**b**) Temperature-induced shift of the Bragg transmission dip. The colored dip curves schematically indicate shifts with increasing temperature.

Figure 1 illustrates the surface-mounted FBG and the temperature-induced displacement of its transmission resonance. The Bragg wavelength is given by

*λ*_B_ = 2*n*_eff_*Λ*, (1)

where *n*_eff_ is the effective refractive index of the guided mode and Λ is the grating period. Temperature changes both quantities through thermo-optic and thermal-expansion effects. Over the calibrated interval, the wavelength shift is expressed as

*Δλ*_B_ = *K*_T_*ΔT*, (2)

where *K*_T_ denotes the measured temperature sensitivity. In transmission, the Bragg resonance appears as a narrow spectral dip whose center wavelength provides the temperature signal. The supplier-specified 3-dB bandwidth of the commercial FBG was ≤0.3 nm, and its reflectivity was ≥85%. A conventional ultraviolet phase-mask-inscribed commercial FBG (ID FBG20240724QQ; Shanghai Xinyi Sensor Technology Co., Ltd., Shanghai, China) was used.

**Figure 2:**
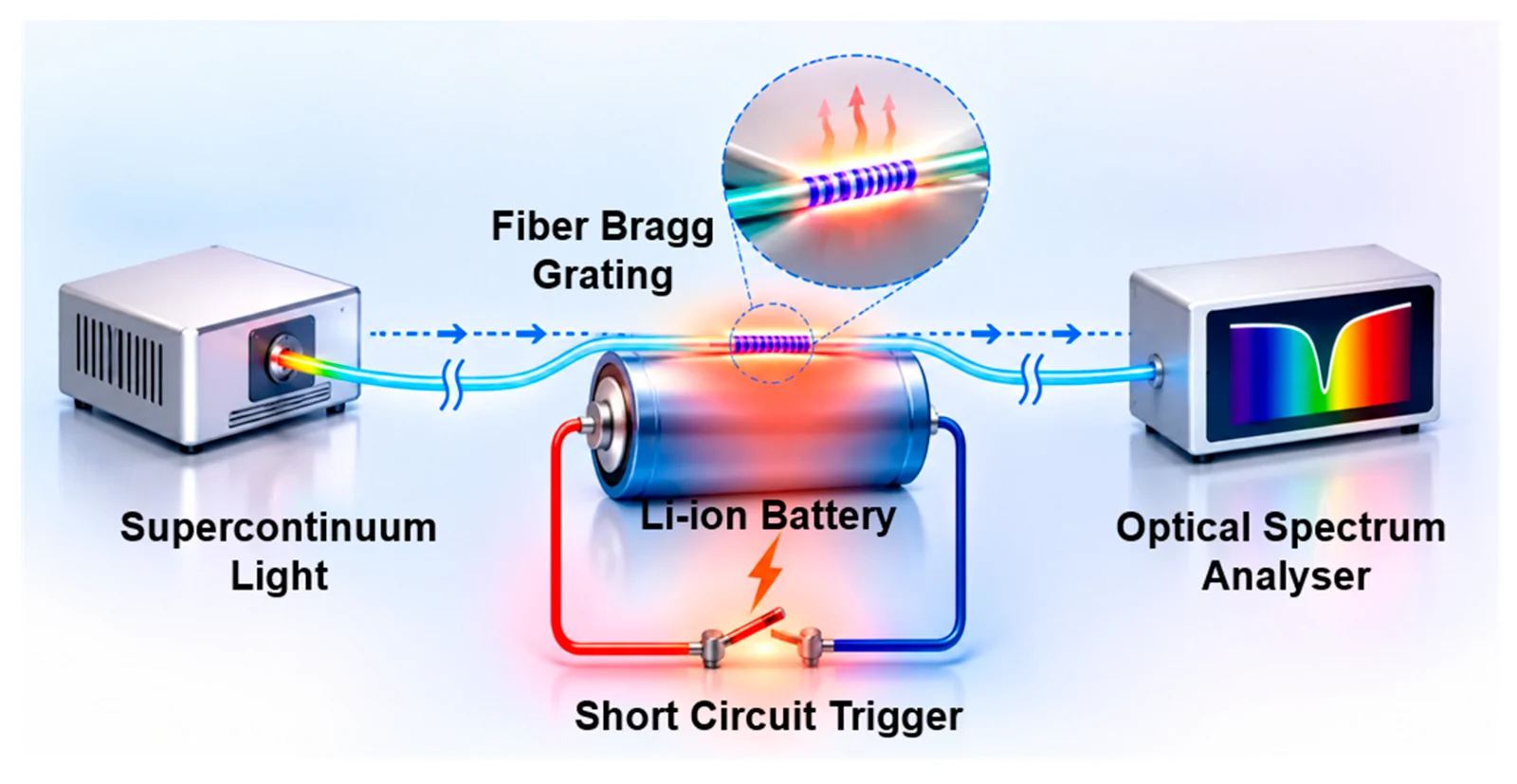
Transmission-mode optical interrogation architecture for battery thermometry. Two 1 km single-mode fiber segments connect the surface-mounted FBG to the remote broadband source and optical spectrum analyzer. Blue arrows indicate optical propagation, while the red connection represents the external short-circuit path.

The interrogation configuration is shown in Figure 2. Broadband light from an Anyang SC-5 supercontinuum source (Wuhan Yangtze Soton Laser Co., Ltd., Wuhan, China; 470–2400 nm) propagates through 1 km of single-mode fiber to a commercial FBG with a nominal center wavelength near 1536 nm. After the grating, a second 1 km single-mode fiber delivers the transmitted spectrum to a Yokogawa AQ6370D optical spectrum analyzer (Yokogawa Test & Measurement Corporation, Tokyo, Japan; 600–1700 nm). The analyzer was operated with a 0.02 nm spectral-resolution setting, a 0.003 nm sampling interval, and an approximately 2 s acquisition time per spectrum. This transmission configuration provides an approximately 2 km optical propagation path while locating only the passive fiber sensor at the battery. The total output power of the SC-5 source was 263.4 mW. The two 1 km leads used YOFC SM28-type single-mode fiber (Yangtze Optical Fibre and Cable Joint Stock Limited Company, Wuhan, China), with a nominal attenuation of approximately 0.2 dB/km near 1550 nm, corresponding to an estimated 0.4 dB over the 2 km link.

### 2.2. Battery Short-Circuit Tests and Cooling Configurations

Commercial NCR18650BD cylindrical lithium-ion cells (Panasonic Energy Co., Ltd., Moriguchi, Osaka, Japan) (nominal capacity: 3100 mAh) were prepared at 100% state of charge; the open-circuit voltage before testing was 4.1 V. The FBG sensing region was placed against a single location on the cell sidewall without rigid bonding, with slack fiber secured using adhesive tape. External short circuit was initiated by directly connecting the positive and negative terminals with welded nickel strips. Under natural air cooling, the experiment took place in laboratory air at approximately 25 °C. Under liquid immersion cooling, a separate cell and FBG were fully immersed in approximately 2000 mL of static commercial No. 10 transformer oil (Aobite, Shenzhen, China), initially near 25 °C. No coolant circulation was applied. The thermophysical properties of mineral insulating oil at 25 °C are listed in the Supplementary Information [51]. Commercial No. 10 transformer oil was selected for electrical insulation and its established use as a heat-transfer medium in power equipment.

The temperature trajectories are plotted against elapsed time from the start of FBG recording, and measured acquisition times and gaps were retained without inserting temperature data. One additional external short-circuit test was performed under each cooling condition; short-circuit current was read from an AiwoSi 828D DC clamp meter (Wuhan Zhongce Hongtu Measuring Instruments Co., Ltd., Wuhan, China) and recorded on video. The current traces use the first clear current rise in each video as their time origin, while the temperature traces use the FBG-recording time base. The open-circuit voltage before each test was recorded, but voltage during the short circuit and external circuit resistance were not measured; the current records therefore characterize part of the fault loading but do not permit a direct calculation of the time-resolved heat-generation rate. Data processing and figure preparation were performed using OriginPro 2021 (OriginLab Corporation, Northampton, MA, USA).

### 2.3. Calibration and Thermal-Response Metrics

For calibration, the FBG was placed in an ECOM LCO 102 DOUBLE column oven (ECOM spol. s r.o., Chrášťany, Czech Republic), and transmission spectra were acquired after 10 min of stabilization at seven temperatures: 25.6, 39.8, 59.8, 69.8, 79.7, 94.9, and 132.3 °C. The Bragg-dip positions were extracted and linearly fitted against the furnace temperature to convert the wavelength shifts into FBG-inferred surface temperature.

To compare records with different initial temperatures, the temperature change is defined as

*ΔT*(*t*) = *T*(*t*) − *T*_0_, (3)

where T_0_ is the initial temperature baseline. For the original tests, it is the mean of the available measurements within the first 30 s; the provisional baselines used for the additional tests are specified in the Supplementary Information. The maximum temperature change is

*ΔT*_max_ = *T*_max_ − *T*_0_, (4)

where *T*_max_ is the highest recorded temperature. The time to peak, t_p_, refers to the FBG-recorded elapsed time at this maximum. For the post-peak comparison, elapsed time is referenced to the respective temperature peak:

*t*^′^ = *t* − *t*_p_, (5)

The corresponding normalized temperature change is

(6) $$\theta \left(t^{\prime }\right)=\frac{T\left(t_{p}+t^{\prime }\right)-T_{0}}{T_{\max}-T_{0}},$$

The post-peak half-decay time, t_0.5_, is the first recorded elapsed time at which θ ≤ 0.5. The cumulative positive temperature change within the common observation window is defined as

(7) $$I_{T}=\int_{0}^{3300\;s}\max\left[\Delta T\left(t\right),0\right]\mathrm{dt}$$

and calculated by trapezoidal integration between the available measurements without adding temperature samples. *I*_T_ characterizes the cumulative surface-temperature excursion above the baseline.

### 2.4. Use of Generative AI Tools

ChatGPT (OpenAI, San Francisco, CA, USA; GPT-6; https://chatgpt.com) was used to assist in the visual presentation of Figure 1 and Figure 2 based on the authors’ original design concepts and the actual experimental configuration. It was also used for English-language polishing. All experimental data were obtained from measurements; no experimental data were generated or modified using generative AI.

## 3. Results

### 3.1. Calibration of the FBG Temperature Response

**Figure 3:**
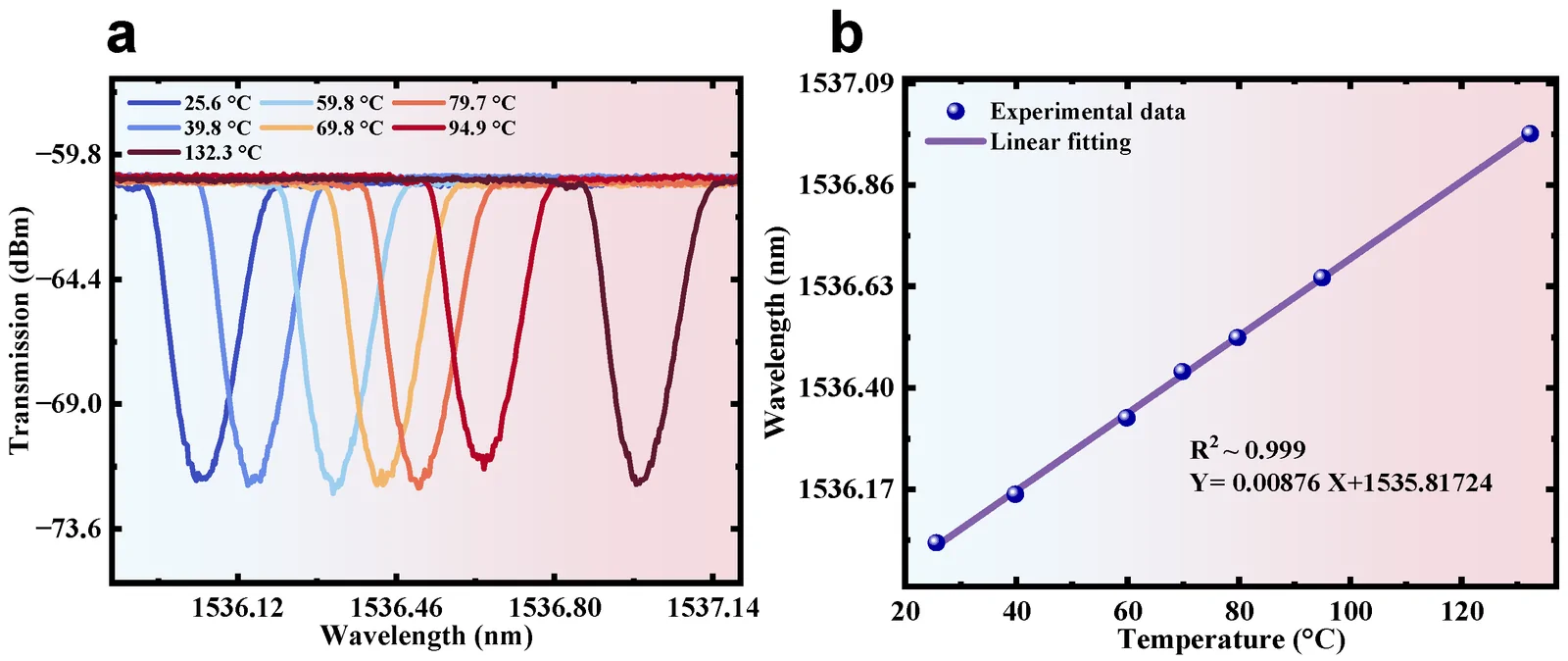
Temperature-dependent Bragg transmission response. (**a**) Measured transmission spectra at seven calibration temperatures. (**b**) Linear relationship between the Bragg wavelength and temperature.

Figure 3a displays the FBG transmission spectra at seven calibration temperatures, including 132.3 °C. The Bragg dip shifts toward longer wavelengths as temperature increases. The seven-point linear fit in Figure 3b yields

*λ*_B_ = 0.00876013*T* + 1535.81724, (8)

where *λ*_B_ is in nm and T is in °C. The measured temperature sensitivity is 8.76 pm °C^−1^ (R^2^ = 0.99955) over 25.6–132.3 °C. This calibration was used to infer the local surface temperature from the Bragg-dip wavelength.

### 3.2. Short-Circuit Heating Under Natural Air Cooling

**Figure 4:**
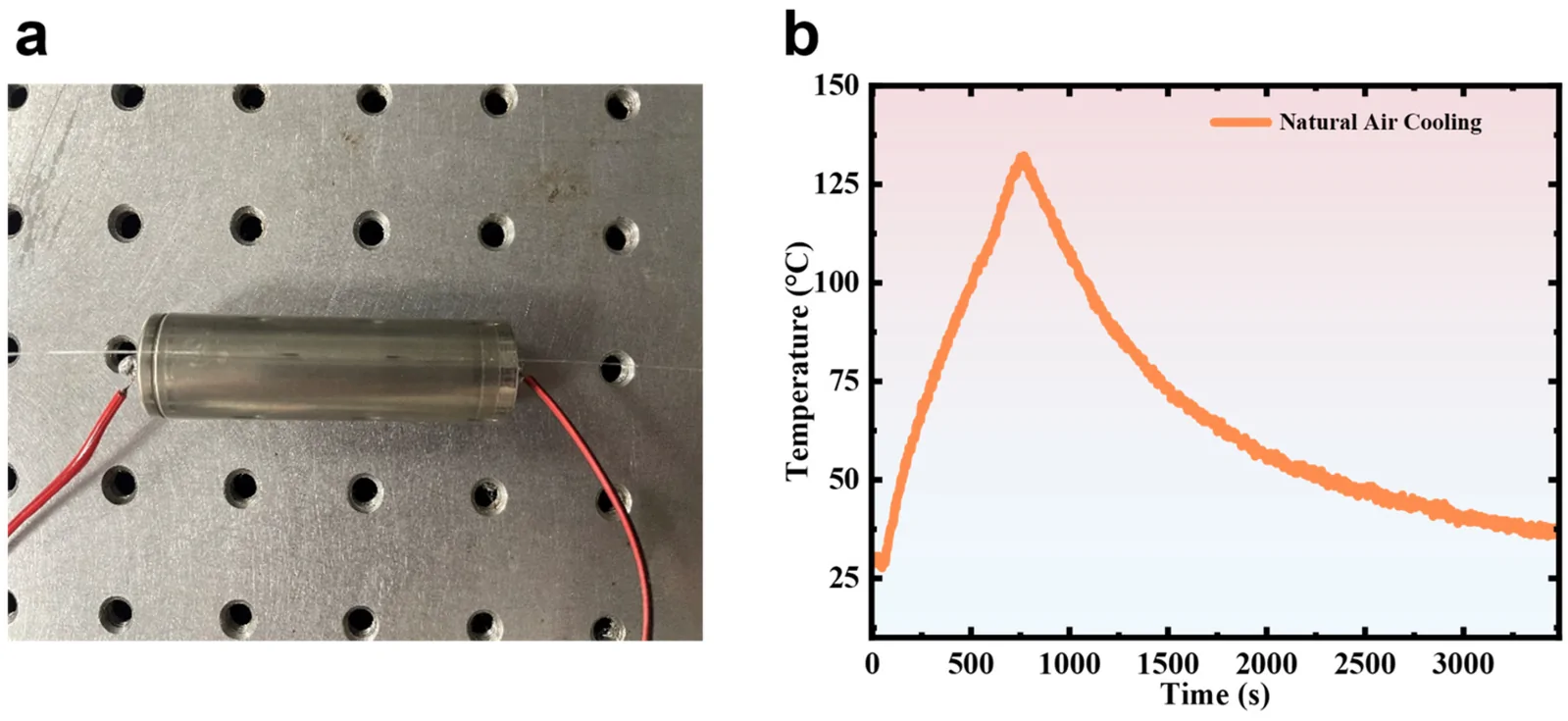
External-short-circuit experiment under natural air cooling. (**a**) Photograph of the test cell and electrical connections. (**b**) FBG-inferred surface-temperature history during heating and subsequent cooling.

The natural air cooling arrangement and its FBG-inferred surface-temperature history from the original test (Trial 1) are presented in Figure 4. The initial temperature was approximately 29.4 °C. The temperature rose to 132.27 °C at an FBG-recorded elapsed time of approximately 769 s, corresponding to a maximum change of 102.87 °C. The subsequent decline was prolonged, indicating persistent elevated surface temperature.

### 3.3. Short-Circuit Thermal Response Under Liquid Immersion Cooling

**Figure 5:**
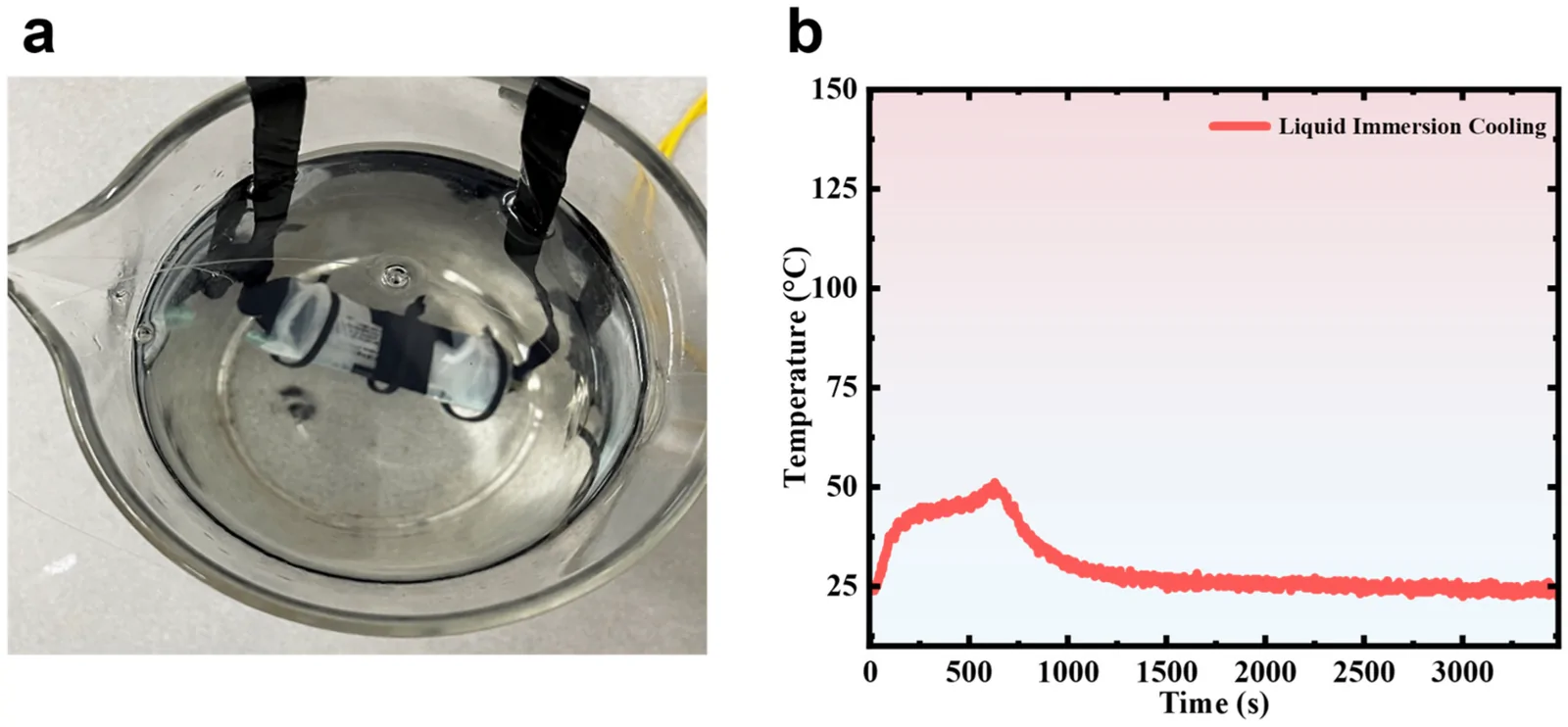
External-short-circuit monitoring under static transformer-oil immersion. (**a**) Photograph of the fully immersed cell and FBG. (**b**) FBG-inferred surface-temperature evolution.

Figure 5 shows the original liquid immersion test (Trial 1), with the cell and FBG fully immersed in static transformer oil. Starting near 24.6 °C, the surface temperature initially rose rapidly and then increased more slowly before reaching 51.12 °C at an FBG-recorded elapsed time of approximately 630 s (maximum rise: 26.53 °C). The recorded temperature subsequently declined toward the initial bath temperature.

### 3.4. Quantitative Comparison of Heating and Cooling Dynamics

**Figure 6:**
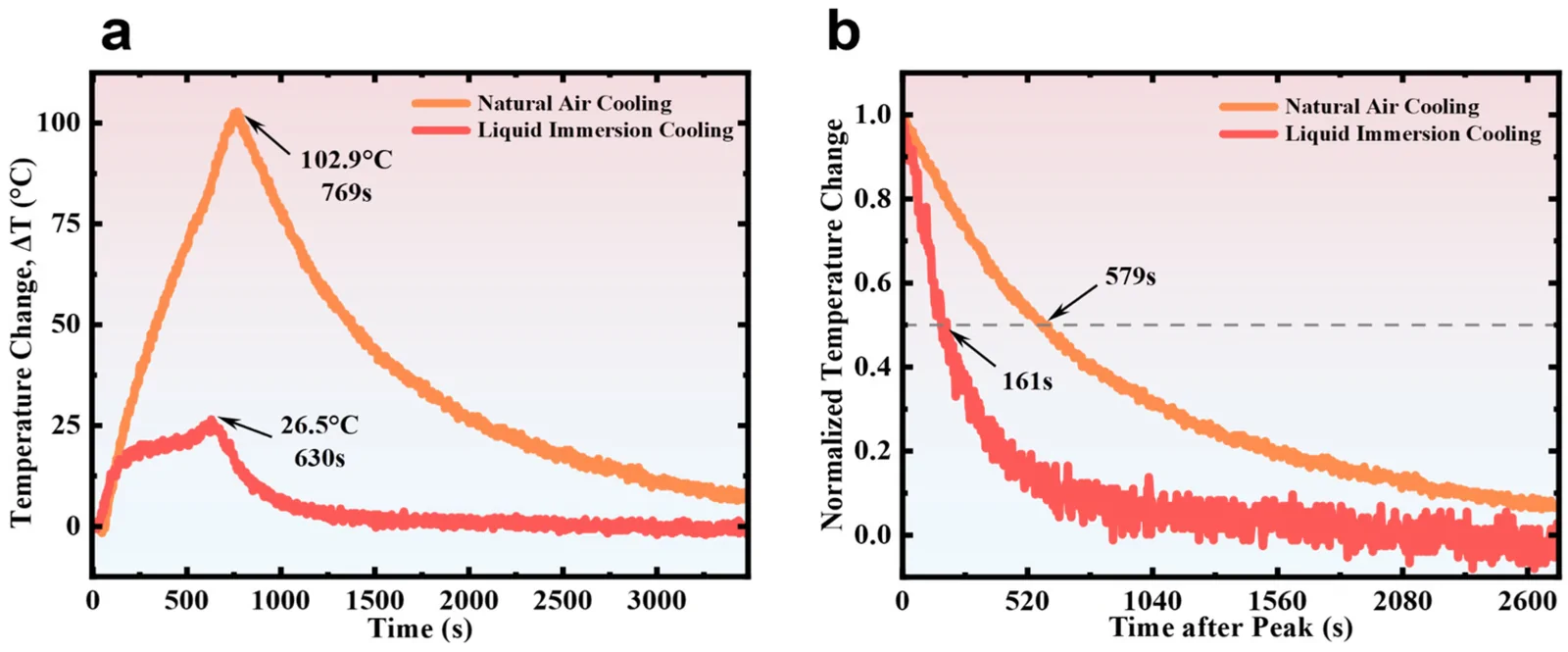
Surface-temperature responses in the original short-circuit tests (Trial 1). (**a**) Temperature rise relative to each test’s baseline. (**b**) Post-peak normalized temperature change versus time after each recorded maximum; the dashed line indicates half the peak temperature excursion. Native acquisition intervals are retained.

Figure 6a compares the original (Trial 1) temperature-rise trajectories under natural air cooling and static oil immersion using each record’s elapsed-time axis. The maximum temperature changes were 102.87 and 26.53 °C, respectively, corresponding to a 74.2% smaller excursion in the immersion test. The measured peaks occurred at elapsed times of 769 and 630 s, respectively; the earlier peak under immersion describes this original test and is not a general result across the additional trials.

Figure 6b compares the original post-peak trajectories normalized by their maximum temperature rises. The first measured half-decay times were 579 s in air and 161 s under immersion. Over the common 0–3300 s record window, the integrated positive temperature changes were approximately 129,216 and 19,091 °C s, respectively (Table 1). In the additional tests (Trial 2), the maximum surface temperatures were 106.34 °C in air and 38.93 °C under immersion, and the first measured post-peak half-decay times were approximately 530 and 265 s, respectively (Figure S2). The corresponding peak currents were 19.46 and 8.40 A (Figure S1), indicating different electrical loading between the two additional tests. The two rounds therefore show the same direction of observed temperature moderation and post-peak recovery under immersion, although the absolute trajectories differ and the Trial 2 current profiles were not matched between cooling conditions.

## 4. Discussion

### 4.1. Heat Generation and Dissipation Under Immersion

The immersed cell exhibited a smaller surface-temperature excursion in the original test. Direct contact with the approximately 2 L static oil bath provides a heat-transfer pathway into the surrounding liquid, while the measured response also reflects internal heat generation and the electrical loading during the short circuit. The additional tests also showed lower surface-temperature excursions and shorter observed post-peak half-decay times under immersion, but the recorded current profiles differed between cooling conditions. The data therefore support a comparison of the tested configurations; they do not isolate the effect of the coolant under matched electrical loading. Because voltage during the short circuit and external circuit resistance were not measured, the time-resolved heat-generation term cannot be quantified from the present measurements.

Previous studies have examined battery temperature regulation using synthetic-ester and static immersion cooling [17,18]. The present measurements add a time-resolved view of the surface-temperature response to an external short circuit under natural air and static transformer-oil immersion, covering the heating stage, peak formation, and post-peak recovery. The comparison is based on the recorded temperature and current trajectories rather than a fitted energy-balance model, because the available measurements do not independently determine the cell-equivalent heat capacity or the effective heat-transfer coefficient for this arrangement. Vikram et al. [19] additionally evaluated transformer-oil immersion for module temperature regulation, whereas Wang et al. [20] and Kannan et al. [21] investigated thermal-runaway and off-gassing under immersion. Those studies help establish the wider experimental context but involve different initiating events and monitored outcomes; the present temperature trajectories alone cannot establish effects on thermal-runaway thresholds or gas emissions.

### 4.2. Evolution of Heating and Formation of the Temperature Peak

The heating trajectories differed between the two environments. In the original tests, air cooling exhibited a sustained increase in surface temperature, whereas the immersion record included a prolonged stage of slower warming. A static oil bath creates a different thermal boundary from laboratory air, and the measured curve reflects both internal heat generation and heat transfer to the surroundings. The slower-warming stage is an observed feature of the original immersion trace; its detailed mechanism cannot be separated with the available measurements.

Peak timing varied between trials. In Trial 1, the immersion trace reached its maximum earlier than the air trace; this ordering was not reproduced in Trial 2. The time to peak should therefore be treated as a test-specific feature and interpreted alongside the evolving current and temperature trajectories.

Electrical and thermal effects are coupled during an external short circuit [8,9,10]. The current traces recorded in Trial 2 provide context for interpreting the corresponding temperature histories because electrical loading can influence both the heating rate and peak timing. The experiments of Dong et al. [11] and Zhang et al. [12] further document the dependence of external-short-circuit thermal responses on electrical fault conditions and test scale. These findings support our cautious interpretation of the unequal Trial 2 current profiles and the absence of a matched-load coolant comparison.

### 4.3. Post-Peak Temperature Recovery and Thermal Exposure

The post-peak half-decay time and the integral of positive temperature rise characterize different aspects of the measured thermal response: the former describes the time required for the normalized temperature rise to fall to half its maximum, whereas the latter reflects the magnitude and duration of the surface-temperature excursion. Both metrics favored the immersed configuration in the original test, and the additional tests showed the same ordering of the observed half-decay times (Figure S2).

These metrics support the conclusion that the tested oil-immersed configuration exhibited a smaller and shorter surface-temperature excursion. The comparison remains limited by two trials per cooling condition, provisional baselines in Trial 2, and different recorded current profiles in the additional tests. A comprehensive measurement-uncertainty budget was not established, so the observed trends should not be interpreted as a statistically generalized, matched-load estimate of coolant-only performance.

### 4.4. Role of Fiber-Optic Thermometry in Immersed Battery Safety Studies

During immersed electrical-abuse tests, the FBG provides continuous surface-temperature monitoring without electrical leads at the measurement point. Its optical readout also avoids the need for a line of sight to the immersed cell, unlike infrared thermography. The same sidewall position was used in both cooling configurations, providing a consistent basis for comparing their surface-temperature trajectories [31,33]. The grating was placed against the cell sidewall without rigid bonding, and slack fiber was secured with adhesive tape to reduce mechanical constraint and strain transfer from cell expansion while maintaining thermal contact. Because no independent strain channel was recorded, any residual strain contribution cannot be quantified; the reported values should therefore be understood as FBG-inferred local surface temperatures under this mounting configuration. Prior in-cell and multi-parameter implementations have achieved temperature–pressure or temperature–strain discrimination through tailored fiber sensing configurations [41,46,47]. Such discrimination was not implemented in our single-FBG surface-contact arrangement, and an independent strain contribution should therefore not be inferred from the present calibration.

The two 1 km optical-fiber leads allow the optical source and spectrum analyzer to remain away from the battery test region. This combination of electrically passive sensing and remote wavelength readout enables monitoring of thermal transients in a static insulating-oil bath and may be extended to multiple sensing points in future studies. Representative earlier battery temperature-sensing studies and the present approach are compared in Table S1 (Supplementary Information).

## 5. Conclusions

This study used a passive FBG to compare local surface-temperature responses to external short circuits under natural air and static transformer-oil immersion. In the original tests, peak temperatures were 132.27 and 51.12 °C, with post-peak half-decay times of 579 and 161 s, respectively. Additional tests again showed a smaller temperature excursion and shorter half-decay time under immersion, although the current profiles differed between conditions.

The results establish the feasibility of long-reach, electrically passive FBG thermometry in immersed battery-abuse tests and support its use for monitoring transient surface-temperature behavior in immersion-cooled battery energy-storage systems. The findings describe the two tested configurations; further replicated tests with synchronized electrical measurements would be needed to quantify uncertainty and separate coolant effects from differences in electrical loading.

## Figures and Tables

**Table 1:** Thermal-response characteristics of the original external-short-circuit tests (Trial 1).

Parameter	Natural Air Cooling	Liquid Immersion Cooling
Initial temperature, T_0_ (°C)	29.40	24.58
Peak temperature, T_max_ (°C)	132.27	51.12
Maximum temperature change, ΔT_max_ (°C)	102.87	26.53
Time to peak, t_p_ (s)	769	630
Mean temperature-rise rate to peak (°C s^−1^)	0.134	0.042
Post-peak half-decay time, t_0.5_ (s)	579	161
Integrated positive temperature change (°C s)	129,216	19,091

Note: T_0_ is the mean of available measurements during the first 30 s of each original test; t_p_ refers to the FBG-recorded elapsed time at the peak; t_0.5_ is the first recorded post-peak time at which the normalized temperature rise is ≤0.5. The temperature integral uses measured points within the common 0–3300 s window.

## Data Availability

The data that support the findings of this study are available from the corresponding author upon reasonable request.

## Supplementary Materials

The following supporting information can be downloaded at: https://www.mdpi.com/article/10.3390/s26216988/s1. Figure S1: Current and FBG-inferred temperature-rise records from the additional (Trial 2) external short-circuit tests. (a) Short-circuit current traces for natural air cooling and liquid immersion cooling. (b) FBG-inferred temperature rise plotted against elapsed FBG-recording time. The current and temperature traces use separate elapsed-time origins. Figure S2: Temperature rise in (a) air and (b) oil immersion for two independent trials; (c) normalized post-peak temperature rise against elapsed time after each measured maximum. Trial 1 is the original experiment; Trial 2 is the additional experiment. Trial 2 baselines are provisional. Table S1: Comparison with representative battery temperature-sensing studies. The supplementary references correspond to references [22,31,33,36,51] in the main reference list.
