# Supplementary material for: Fiber Bragg Grating Monitoring of Thermal Dynamics in Immersion-Cooled Lithium-Ion Batteries
Source: Sensors (Basel). 2026 Nov 5;26(21):6988. doi: 10.3390/s26216988 (PMC13766418; doi:10.3390/s26216988)
Supplement: Supplementary file 1 [file sensors-26-06988-s001.zip › sensors-4648504-supplementary.pdf]

Fiber Bragg Grating Monitoring of Thermal Dynamics in Immersion-Cooled Lithium-Ion Batteries

Supplementary Experimental Details

Two experimental rounds are presented. Trial 1 corresponds to the original tests reported in the main manuscript; Trial 2 corresponds to the additional tests conducted during revision. In Trial 2, short-circuit current was read from a DC clamp-meter display recorded on video. The current traces use the first clear rise in each video as their time origin, while the FBG temperature traces use elapsed time from the start of FBG recording.

For Trial 1, the baseline T_0 is the mean of available temperatures within the first 30 s. For Trial 2, provisional baselines of 23.72 °C (air, first 13 measured spectra) and 23.41 °C (immersion, first 4 measured spectra) were used. Temperature rise is $\Delta T = T - T_0$, and normalized post-peak change is $\theta = \Delta T/\Delta T_{max}$, with time referenced to each recorded maximum.

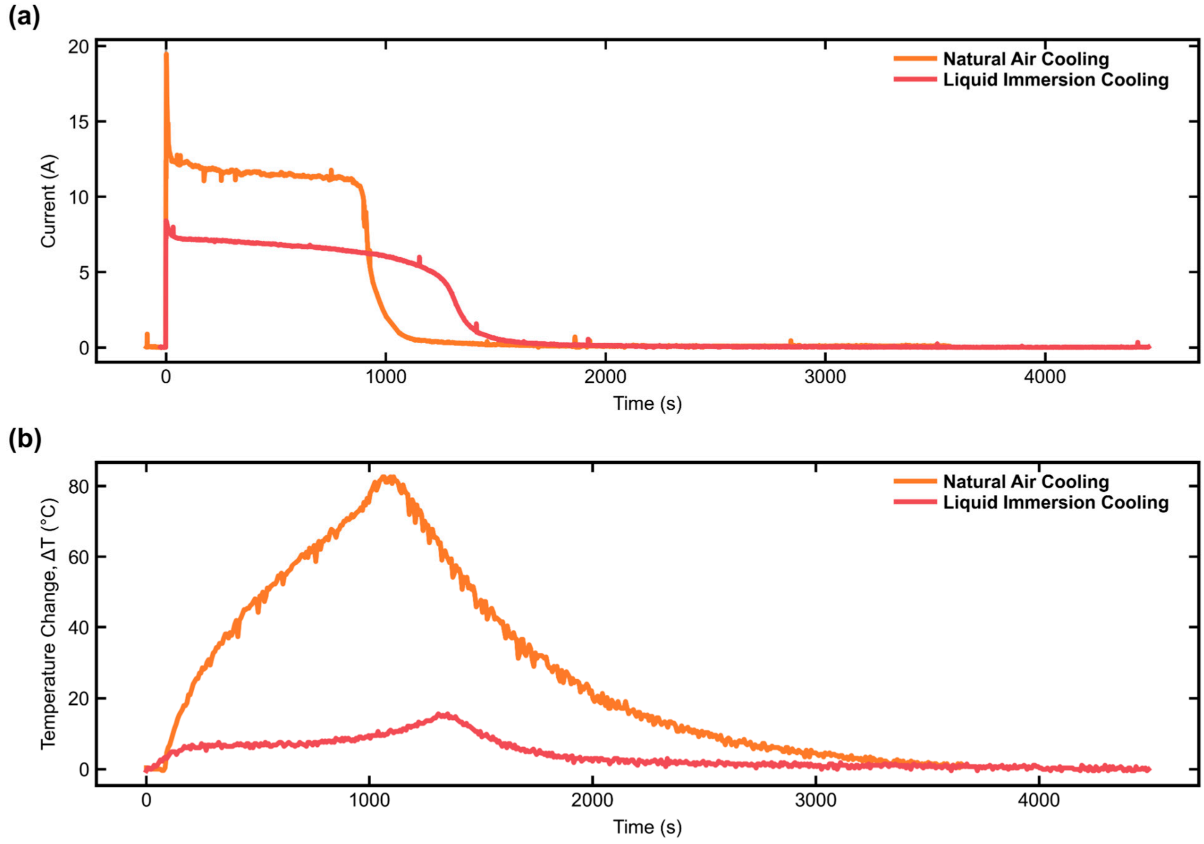

Figure S1. Current and FBG-inferred temperature-rise records from the additional (Trial 2) external short-circuit tests. (a) Short-circuit current traces for natural air cooling and liquid immersion cooling. (b) FBG-inferred temperature rise plotted against elapsed FBG-recording time. The current and temperature traces use separate elapsed-time origins.

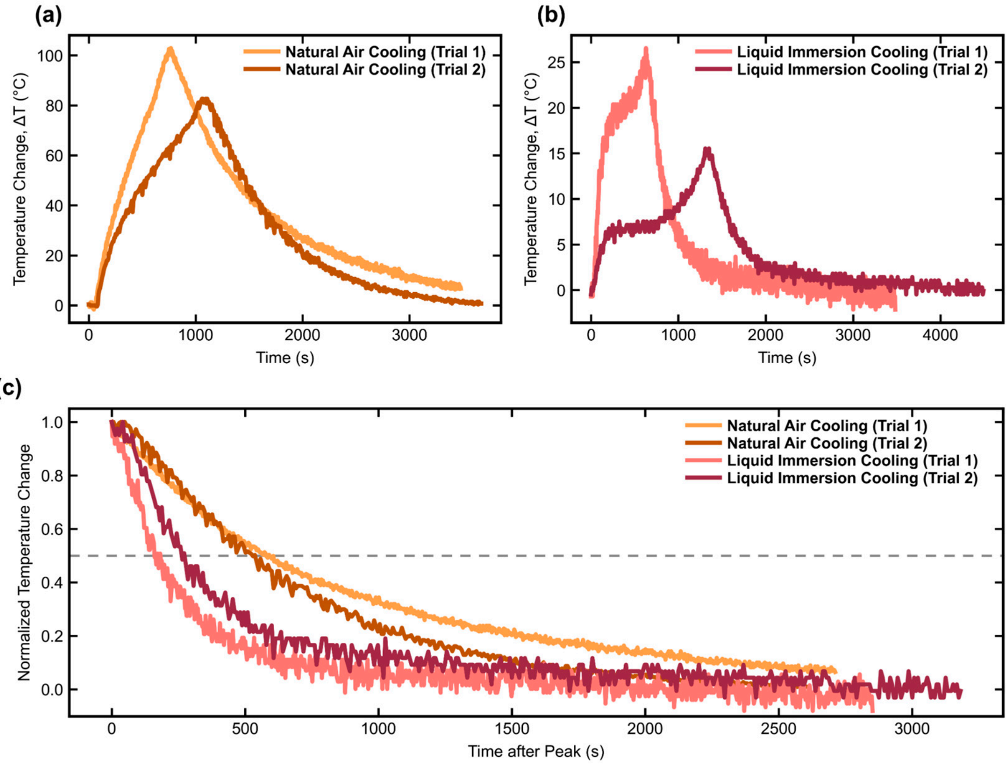

Figure S2. Temperature rise in (a) air and (b) oil immersion for two independent trials; (c) normalized post-peak temperature rise against elapsed time after each measured maximum. Trial 1 is the original experiment; Trial 2 is the additional experiment. Trial 2 baselines are provisional.

Table S1. Comparison with representative battery temperature-sensing studies.

Study	Temperature sensing	Test context	Main focus
Koshkouei et al. [S1]	Thermocouples and IR	Cell cycling	Probe selection, response time and magnetic-field effects
Yang et al. [S2]	FBG	Battery operation	In-situ battery temperature tracking
Nascimento et al. [S3]	FBG and thermocouples	Charge/discharge tests	Direct comparison of temperature-sensor responses
Kim et al. [S4]	Remote FBG–OFDR	Rotating Li-ion cell	Remote and distributed fiber-optic thermometry
This work	Passive transmission-mode FBG; two 1 km leads	External short circuit in air and static oil	Full temperature transient and post-peak recovery under different cooling conditions

The studies differ in test conditions; the comparison highlights research scope rather than claiming a direct sensor-performance benchmark.

Thermophysical Properties of the Immersion Medium

The static bath contained approximately 2000 mL of commercial No. 10 transformer oil (Aobite, Shenzhen, China). At 25 °C, the mineral insulating-oil properties [S5] are density 867 kg m^{-3}, specific heat capacity 1902 J kg^{-1} K^{-1}, thermal conductivity 0.133 W m^{-1} K^{-1}, kinematic viscosity 17.1 mm^2 s^{-1}, and thermal expansion coefficient 7.5×10^{-4} K^{-1}.

References

[S1] Koshkouei, M.J.; Saniee, N.F.; Barai, A. Thermocouple selection and its influence on temperature monitoring of lithium-ion cells. J. Energy Storage 2024, 92, 112072. https://doi.org/10.1016/j.est.2024.112072.
[S2] Yang, G.; Leitão, C.; Li, Y.; Pinto, J.; Jiang, X. Real-time temperature measurement with fiber Bragg sensors in lithium batteries for safety usage. Measurement 2013, 46, 3166–3172.
[S3] Nascimento, M.; Ferreira, M.S.; Pinto, J.L. Real time thermal monitoring of lithium batteries with fiber sensors and thermocouples: A comparative study. Measurement 2017, 111, 260–263. https://doi.org/10.1016/j.measurement.2017.07.049.
[S4] Kim, J.; Lee, S.; Lee, T.; Yoo, J. A remote FBG-OFDR thermometry sensor for EV energy systems. Measurement 2023, 221, 113457. https://doi.org/10.1016/j.measurement.2023.113457.
[S5] Nadolny, Z. Evaluation of Thermal Properties of Various Insulating Liquids Used in Power Transformers. Energies 2024, 17, 3037. https://doi.org/10.3390/en17123037.
